# Supplementary figures and images for: Multi-genome comparisons reveal gain-and-loss evolution of anti-Mullerian hormone receptor type 2 as a candidate master sex-determining gene in Percidae
Source: BMC Biol. 2024 Jun 26;22:141. doi: 10.1186/s12915-024-01935-9 (PMC11209984; doi:10.1186/s12915-024-01935-9)

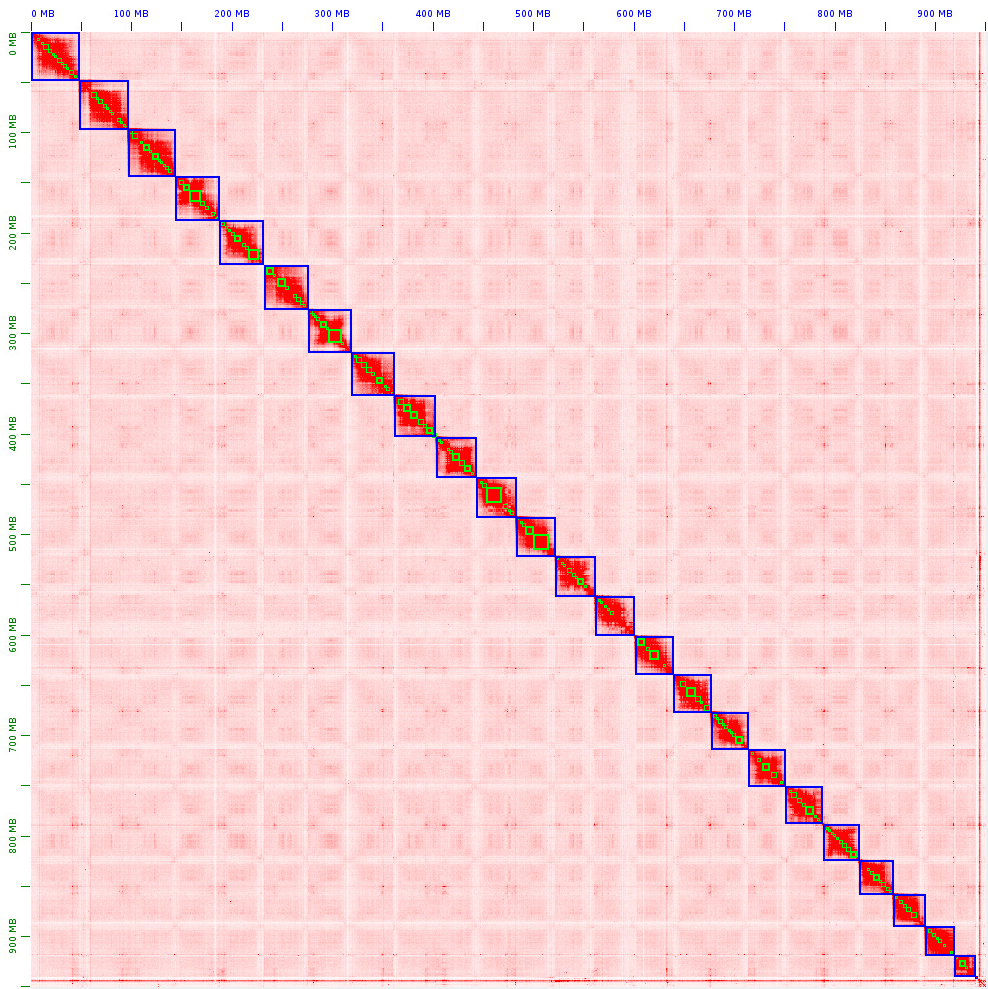

Supplement: Supplementary file 1 — Additional file 1: Fig. S1. Hi-C map of P. fluviatilis genome [file 12915_2024_1935_MOESM1_ESM.png]

*Perca schrenkii*

17

female

8

male

700 bp

500 bp

100 bp

50 bp

*amhr2b* (637bp)

*amhr2a* (523bp)

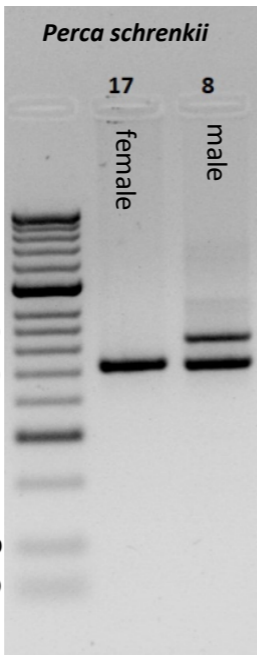

Supplement: Supplementary file 3 — Additional file 3: Fig. S2. A sex-specific 637 bp PCR product amplifies in P. schrenkii male (sample id = 8), while it is absent in female (sample id = 17). The corresponding primer pair also works for sexing of P. flavescens. [file 12915_2024_1935_MOESM3_ESM.pdf]

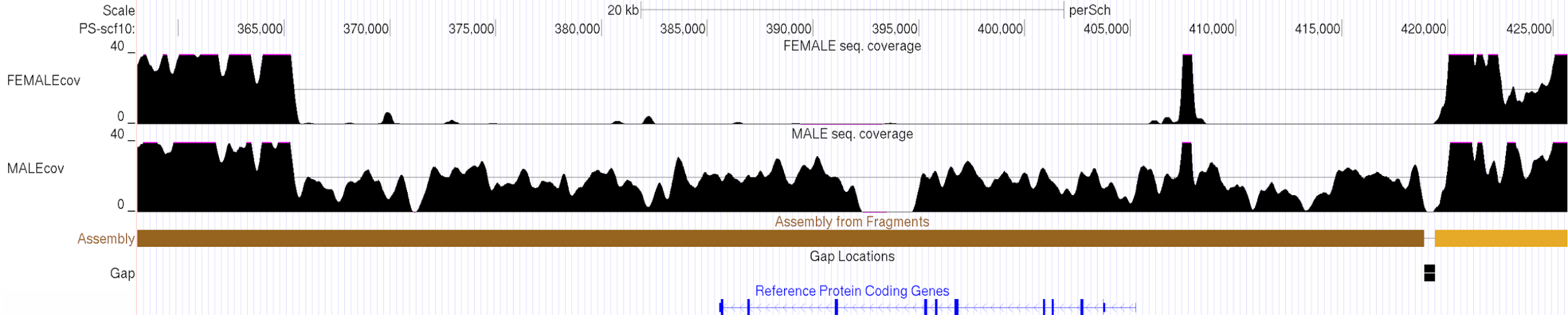

Supplement: Supplementary file 4 — Additional file 4: Fig. S3. Sex-specific sequencing coverage of amhr2b locus in Perca schrenkii in region PS-scf10/CM046795.1: 366,025-418,970. A female and a male genome were sequenced to approximately 40x coverage using short-read sequencing. After filtering for unique mapping reads (mapping quality 60), a clear coverage difference between females and males is visible. The ~53 kbp region has virtually no coverage in females. In contrast males exhibit haploid coverage (about 20x), which is in accordance with a X/Y SD system. [file 12915_2024_1935_MOESM4_ESM.pdf]

Number of females

30  
25  
20  
15  
10  
5  
0

0

5

10

15

20

25

30

35

Number of males

Sequences

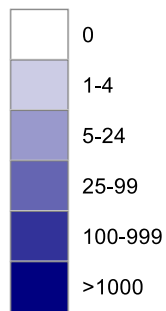

Signif.

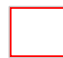

Supplement: Supplementary file 5 — Additional file 5: Fig. S4. A single RAD sex-specific marker is significantly associated with male sex in P. fluviatilis. Tile plot of the distribution of RADSex markers between Perca fluviatilis males (horizontal axis) and females (vertical axis) with a minimum read depth of 1 (d = 1). Color intensity (see color legend on the right) indicates the number of markers present for each of the corresponding number of males and females. A single significant marker at the lower right of the grid was present in all 35 males and absent from all 34 females and is boxed with a red border (Chi-squared test, p < .05 after Bonferroni correction). [file 12915_2024_1935_MOESM5_ESM.pdf]

# Expression in gonads for genes within the *P. fluviatilis* sex-specific region

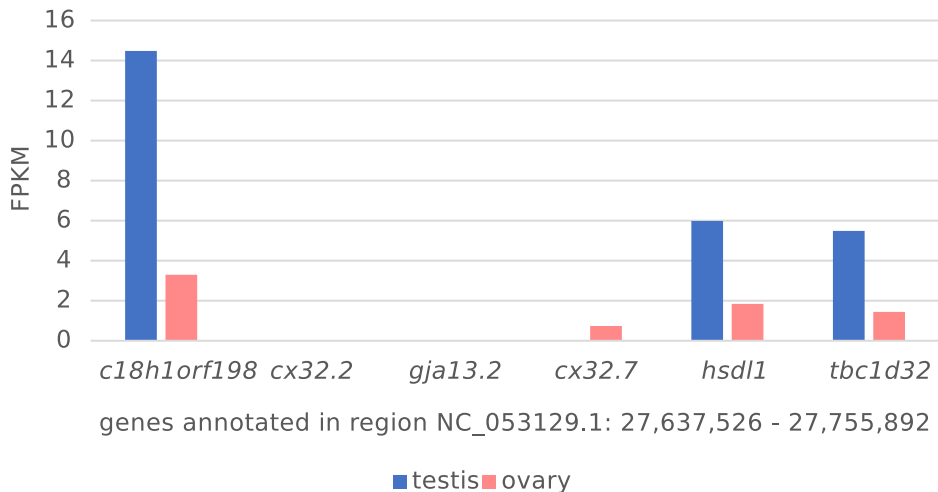

Supplement: Supplementary file 6 — Additional file 6: Fig. S5. Expression of hsdl1 and neighboring genes in public RNAseq datasets (testis: SRR14461526 [81], ovary: SRR14461527 [82]; age of both sampled individuals 9 month). Here hsdl1 expression in testis is 3.25-fold higher than in ovary, for tbc1d32 and c18h1orf198 the ratio is 3.83 and 4.41, respectively. [file 12915_2024_1935_MOESM6_ESM.pdf]

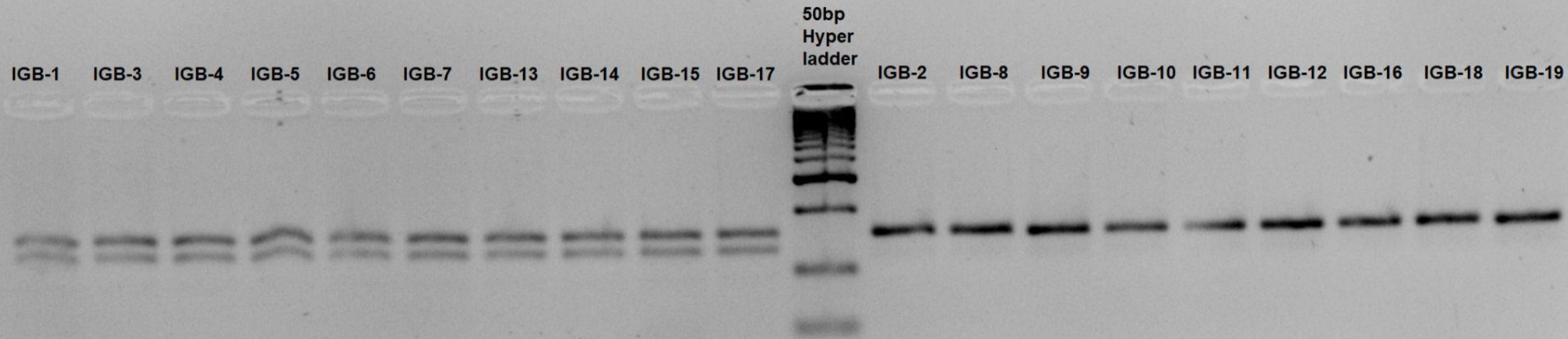

Supplement: Supplementary file 7 — Additional file 7: Fig. S6. Sexing of P. fluviatilis using a 27 bp male specific deletion in Intron 3 of the hsdl1 gene (10 males (left) and 9 females (right) from Lake Mueggelsee, Berlin. The simultaneous amplification in both males and females of the X allele without the 27 bp deletion provides an internal control for this PCR. All XY male samples (N = 10) produce two amplicons due to the small size difference of the X and Y amplified alleles, and all XX females (N = 9) produced only the larger X amplicon. This hsdl1 intronic indel variant is located near the variant SNV5 (distance < 1.5 kbp). [file 12915_2024_1935_MOESM7_ESM.pdf]

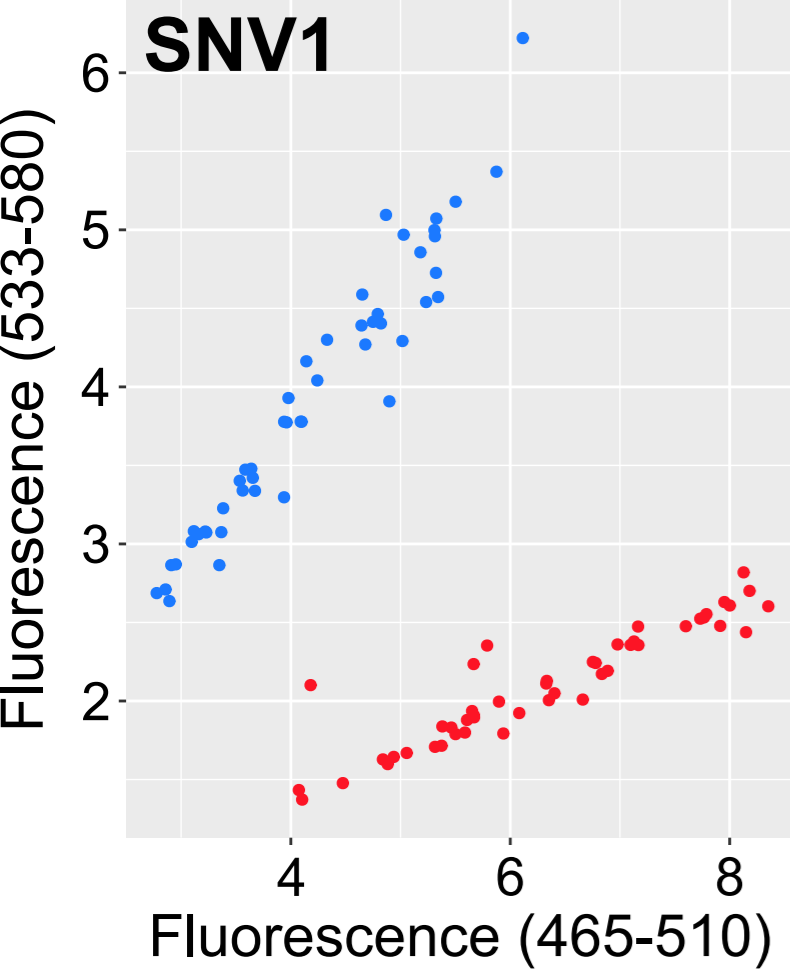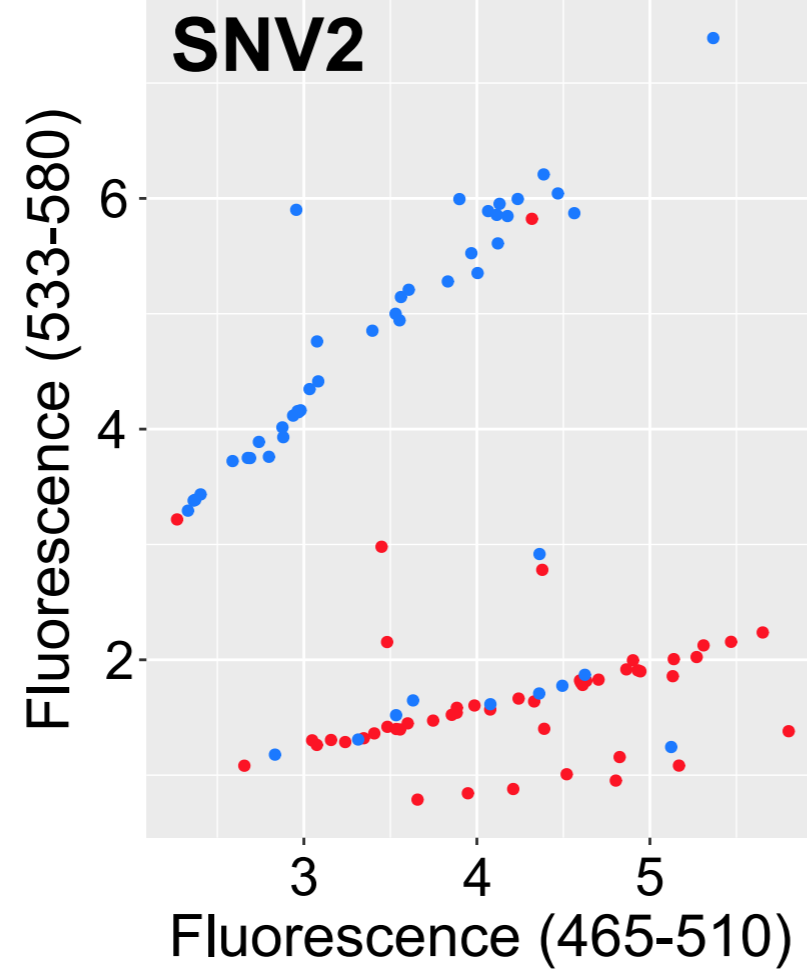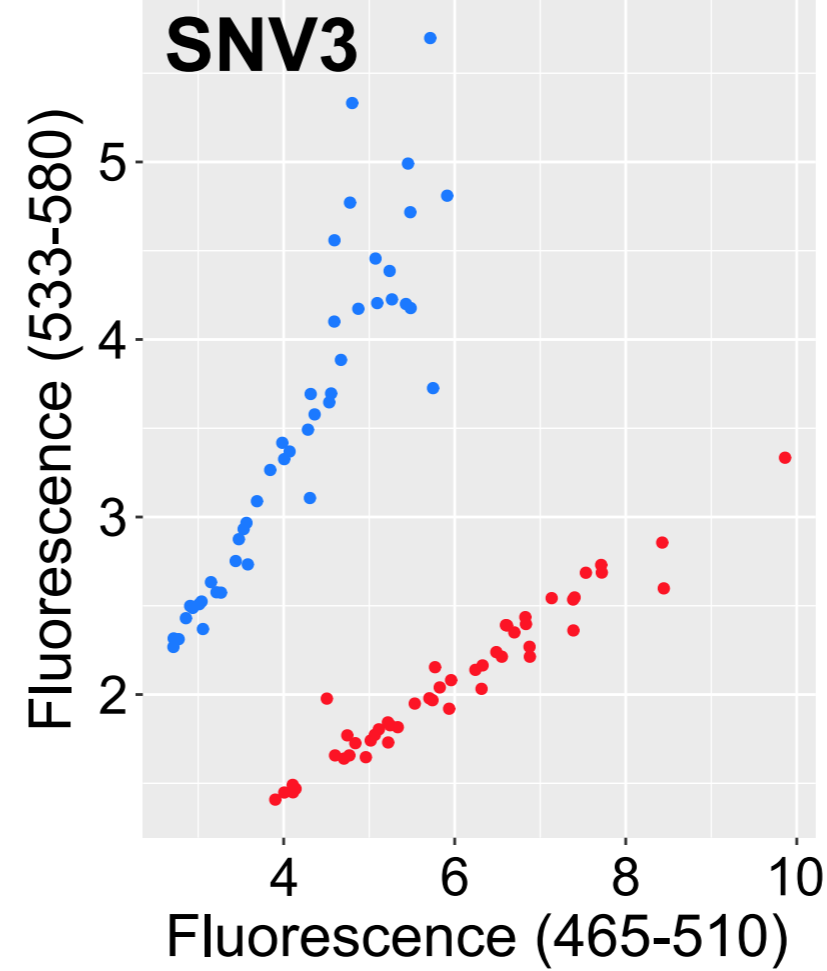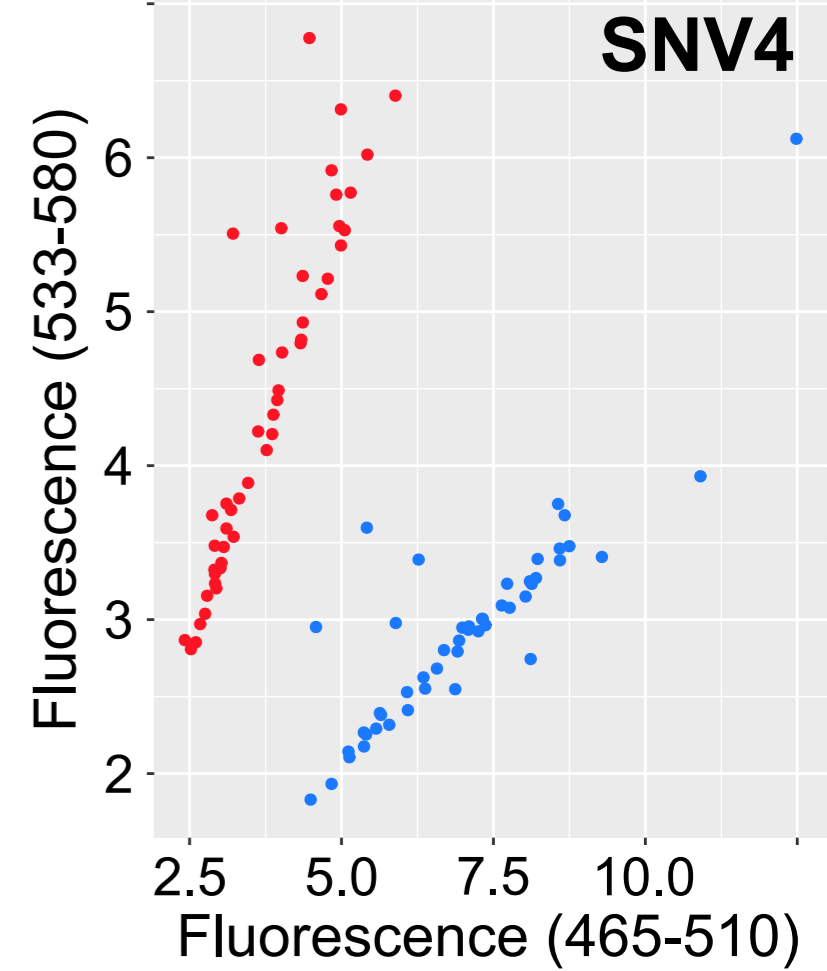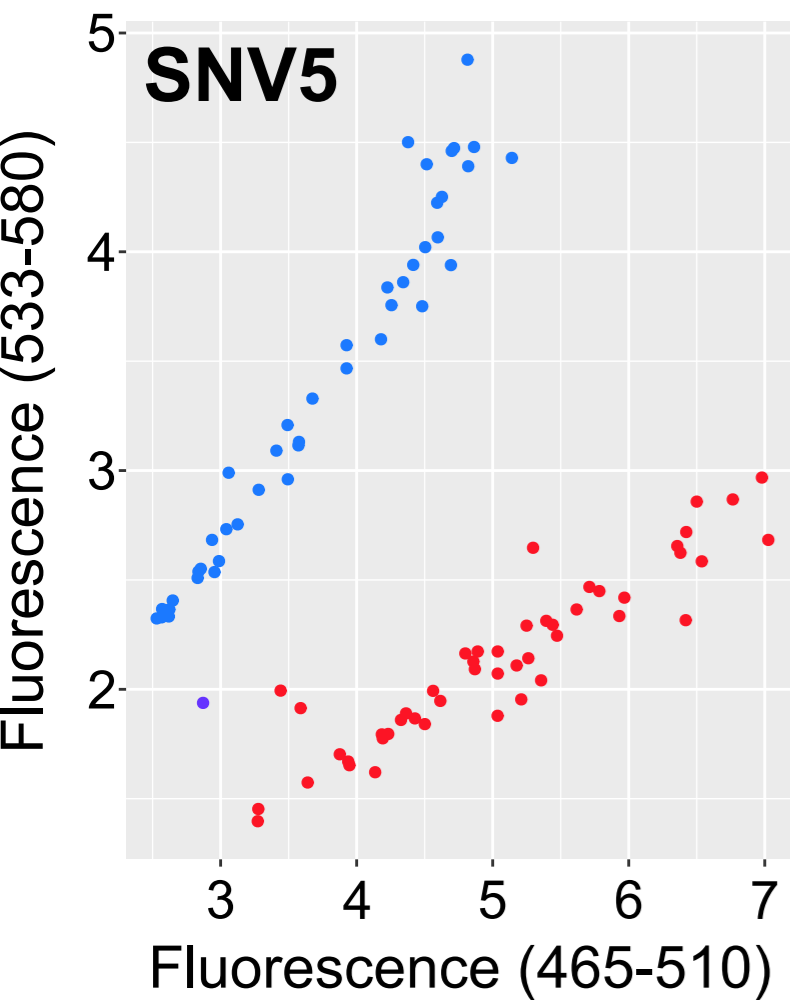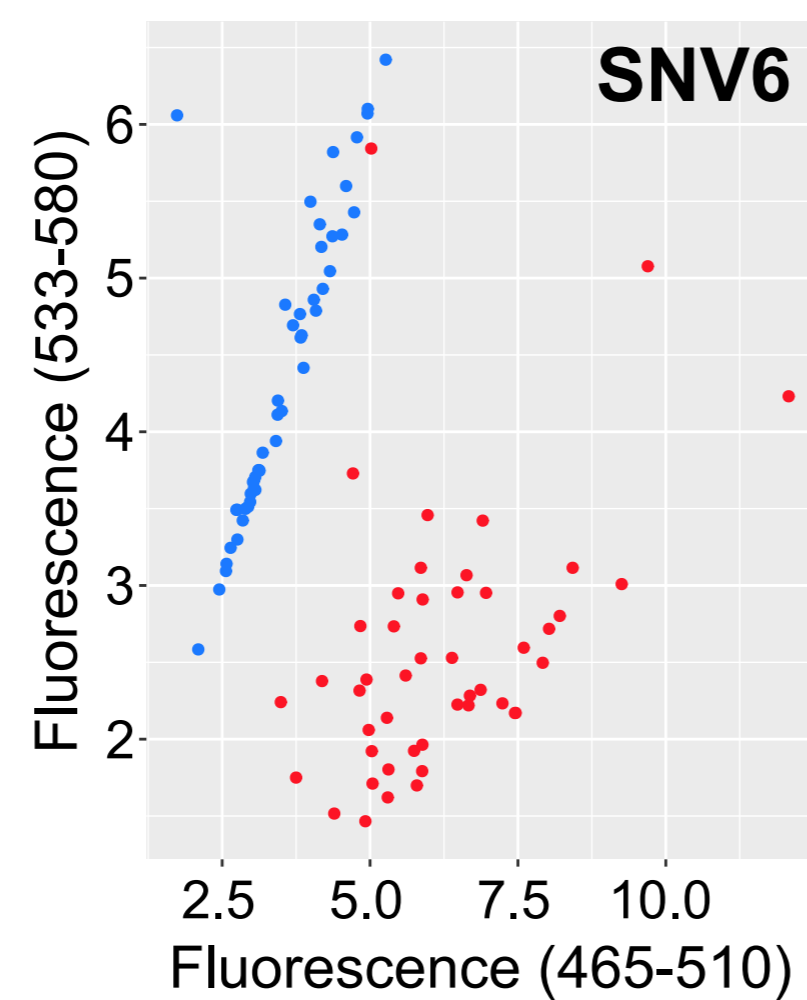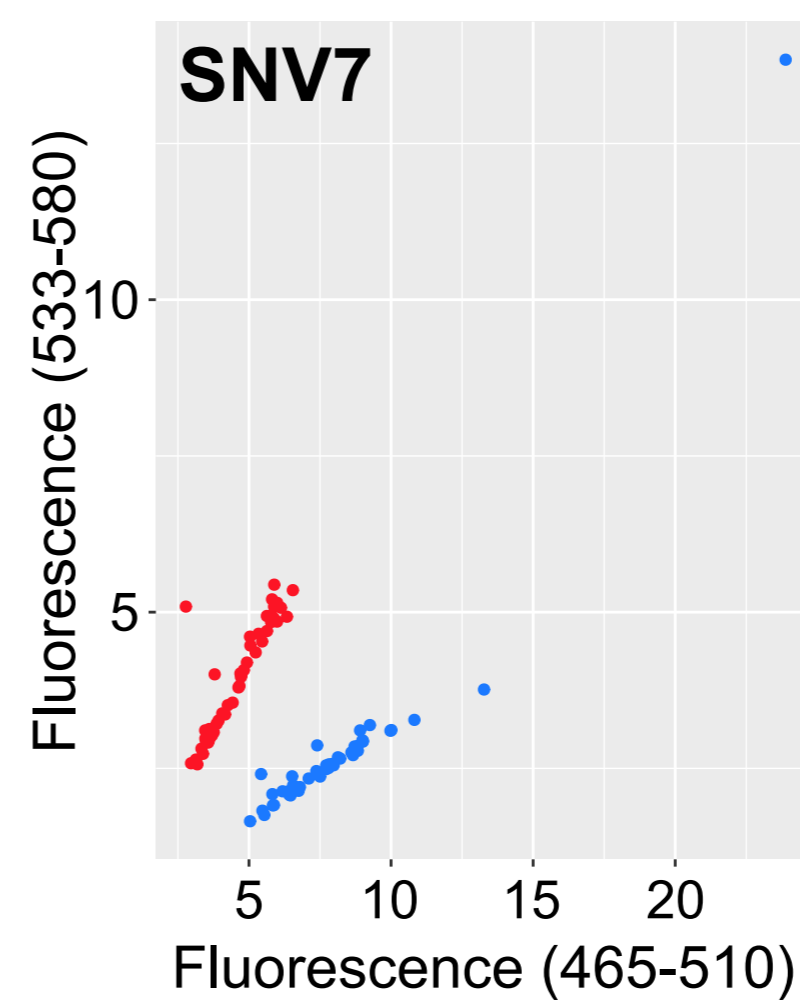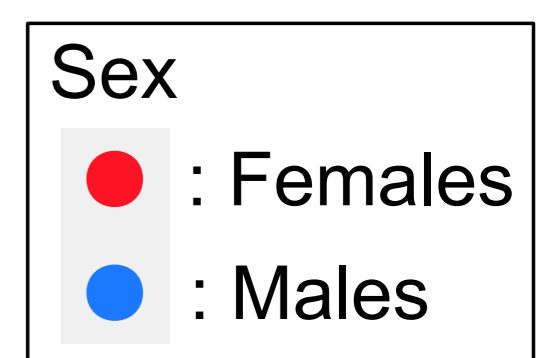

Supplement: Supplementary file 8 — Additional file 8: Fig. S7. KASpar allele-specific PCR assays on seven single sex-specific nucleotide variations (SNV ID#) in P. fluviatilis. For each Single Nucleotide Variation (SNV), primer AL1 was coupled to FAM fluorescent dye and primer AL2 was coupled to VIC fluorescent dye and the end-point fluorescence of these two fluorescent dyes was respectively on the x­ and y­ axes. Male individuals are represented by blue dots and females by red dots. Primers used for analysis can be found in Table 1. [file 12915_2024_1935_MOESM8_ESM.pdf]

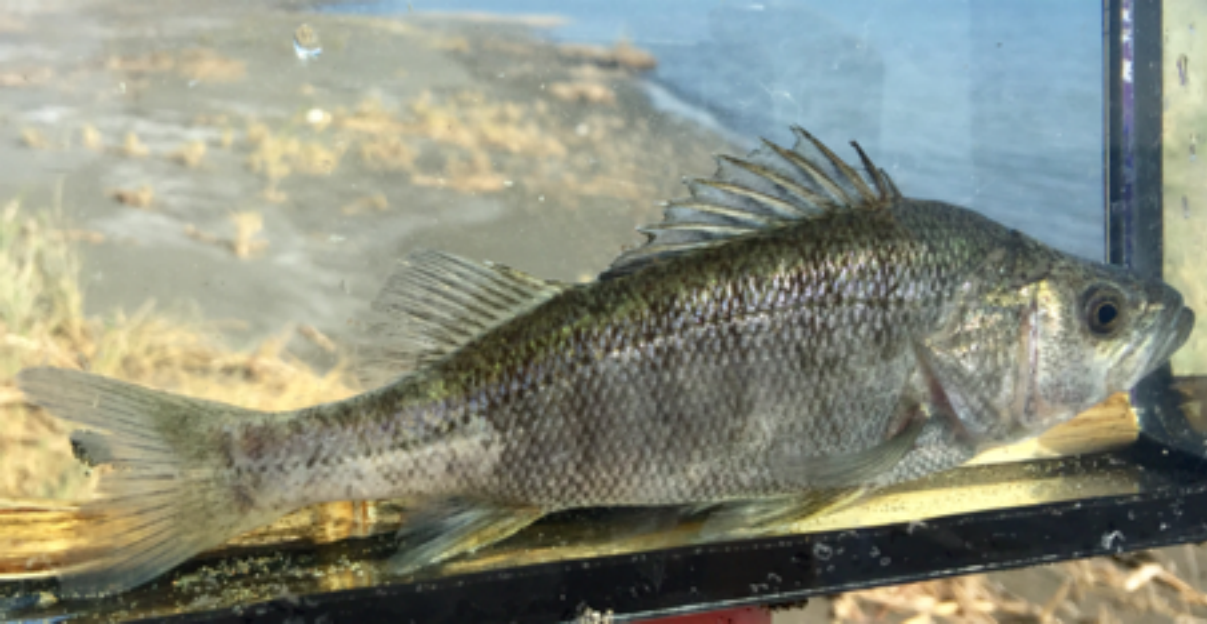

Supplement: Supplementary file 9 — Additional file 9: Fig. S8. Photo-voucher of the male Perca schrenckii sampled at Lake Alakol on 11 September 2018; Kazakhstan (46.328 N, 81.374 E). Photo: Matthias Stöck. [file 12915_2024_1935_MOESM9_ESM.pdf]
