# Supplementary material for: Multi-genome comparisons reveal gain-and-loss evolution of anti-Mullerian hormone receptor type 2 as a candidate master sex-determining gene in Percidae
Source: BMC Biol. 2024 Jun 26;22:141. doi: 10.1186/s12915-024-01935-9 (PMC11209984; doi:10.1186/s12915-024-01935-9)
Supplement: Supplementary file 2 — Additional file 2: Table S1. Annotated repeats in Perca sp. and Sander sp. genomes (RepeatModeler de novo analysis). Repeat elements mentioned in the manuscript have grey highlighting. The class “DNA” is assigned to repeat elements that harbor signals of transposases, but miss further signals to classify them with more detail. [file 12915_2024_1935_MOESM2_ESM.docx]

**Table S1:** Annotated repeats in *Perca sp.* and *Sander sp.* genomes (RepeatModeler *de novo* analysis). Repeat elements mentioned in the manuscript have grey highlighting. The class “DNA” is assigned to repeat elements that harbor signals of transposases, but miss further signals to classify them with more detail.

|  | ***P. fluviatilis*** | ***P. flavescens*** | ***P. schrenkii*** | ***S. lucioperca*** | ***S. vitreus*** |
| --- | --- | --- | --- | --- | --- |
| **length (no gaps)** | 951.053.269 | 877.041.836 | 893.440.234 | 901.028.039 | 785.350.625 |
| **Repeat class** | **bp masked** | **bp masked** | **bp masked** | **bp masked** | **bp masked** |
| DNA | **22.711.613** | 20.520.179 | **23.132.743** | **19.957.261** | 15.428.015 |
| Academ | 2.340.071 | 1.619.886 | 1.842.836 | 2.412.073 | 1.790.843 |
| CMC-Chapaev-3 | 230.562 | 0 | 0 | 0 | 0 |
| CMC-EnSpm | **9.118.071** | 7.588.794 | **4.039.674** | **5.928.087** | 3.520.268 |
| Crypton | 841.031 | 547.307 | 748.707 | 772.498 | 402.911 |
| Crypton-V | 11.212 | 0 | 0 | 0 | 0 |
| Dada | 0 | 33.030 | 206.119 | 0 | 0 |
| Ginger | 0 | 15.288 | 0 | 0 | 0 |
| IS3EU | 1.282.033 | 531.773 | 496.067 | 288.665 | 509.614 |
| Kolobok-Hydra | 0 | 648.047 | 0 | 0 | 70.720 |
| Kolobok-T2 | 1.252.506 | 1.976.511 | 2.668.034 | 1.586.132 | 1.222.046 |
| MULE-MuDR | 251.959 | 1.716.319 | 441.114 | 266.625 | 27.741 |
| Maverick | 2.187.699 | 1.402.756 | 266.487 | 1.342.717 | 0 |
| Merlin | 0 | 0 | 0 | 122.531 | 94.494 |
| Novosib | 0 | 0 | 0 | 0 | 82.199 |
| P | 467.516 | 442.149 | 411.688 | 765.209 | 555.234 |
| PIF-Harbinger | 12.989.790 | 12.205.497 | 12.181.221 | 12.830.055 | 8.037.073 |
| PIF-ISL2EU | 0 | 235.589 | 464.262 | 1.597.340 | 914.106 |
| PiggyBac | **3.844.887** | 2.011.288 | **2.742.549** | **3.776.961** | 2.299.194 |
| Sola | 1.885.532 | 229.560 | 163.435 | 77.871 | 0 |
| TcMar | 17.070 | 135.873 | 0 | 185.840 | 137.413 |
| TcMar-Fot1 | 0 | 30.498 | 0 | 44.754 | 37.051 |
| TcMar-ISRm11 | 4.570.634 | 3.171.866 | 2.722.899 | 2.158.133 | 1.830.923 |
| TcMar-Stowaway | 220.092 | 98.992 | 114.404 | 65.697 | 0 |
| TcMar-Tc1 | 15.259.371 | 12.137.252 | 12.302.578 | 6.557.230 | 10.225.920 |
| TcMar-Tc2 | 627.258 | 465.068 | 299.455 | 227.802 | 468.716 |
| Zator | 0 | 119.597 | 0 | 0 | 0 |
| Zisupton | 30.281 | 0 | 127.852 | 0 | 104.202 |
| Zisupton-hAT-hybrid | 0 | 0 | 0 | 0 | 339.346 |
| hAT | **9.251.059** | 7.244.672 | **9.113.922** | **8.460.295** | 6.356.798 |
| hAT-Ac | 26.161.464 | 27.815.352 | 29.153.525 | 30.578.355 | 27.361.032 |
| hAT-Blackjack | 1.252.051 | 459.611 | 338.327 | 514.383 | 437.930 |
| hAT-Charlie | **7.578.170** | 5.645.728 | **8.825.006** | **5.557.301** | 3.893.733 |
| hAT-Tip100 | 3.892.297 | 2.083.169 | 5.060.148 | 2.183.415 | 2.136.454 |
| hAT-Tol2 | 7.383.933 | 7.130.470 | 10.507.431 | 6.788.875 | 4.948.115 |
| hAT-hAT5 | 1.008.628 | 1.141.196 | 1.066.203 | 873.439 | 560.575 |
| hAT-hAT6 | 153.938 | 129.767 | 0 | 0 | 37.338 |
| hAT-hobo | 693.242 | 589.992 | 435.263 | 245.056 | 840.680 |
| LINE | 253.345 | 0 | 0 | 135.963 | 162.701 |
| CR1 | 61.557 | 52.996 | 46.593 | 68.770 | 58.461 |
| Dong-R4 | 0 | 161.938 | 0 | 0 | 0 |
| I | 902.480 | 1.009.950 | 1.721.862 | 451.473 | 448.921 |
| I-Nimb | 187.474 | 66.650 | 179.364 | 0 | 89.737 |
| Jockey | 1.281.097 | 0 | 0 | 249.376 | 0 |
| L1 | 3.161.932 | 2.720.443 | 3.146.061 | 3.530.190 | 3.456.430 |
| L1-Tx1 | 1.332.071 | 707.047 | 492.758 | 1.328.940 | 247.067 |
| L2 | **24.295.832** | 18.778.236 | **18.931.729** | **15.671.956** | 10.981.470 |
| Penelope | 696.540 | 651.596 | 168.974 | 978.870 | 436.687 |
| Proto2 | 432.031 | 406.555 | 335.607 | 288.595 | 210.869 |
| R1 | 0 | 0 | 0 | 146.213 | 277.081 |
| R2-Hero | 258.797 | 37.021 | 152.400 | 101.778 | 204.860 |
| R2-NeSL | 139.492 | 0 | 191.055 | 106.574 | 62.871 |
| RTE | 1.012.896 | 0 | 0 | 0 | 0 |
| RTE-BovB | 3.915.100 | **11.058.368** | 4.053.280 | 5.607.772 | **8.522.404** |
| RTE-RTE | 0 | 0 | 0 | 169.573 | 0 |
| RTE-RTEX | 0 | 26.699 | 19.406 | 32.367 | 0 |
| RTE-X | 738.450 | 801.862 | 1.308.994 | 1.325.349 | 418.476 |
| Rex-Babar | **8.447.394** | 7.183.710 | **11.161.891** | **7.936.953** | 5.965.113 |
| Tad1 | 0 | 0 | 0 | 138.056 | 320.275 |
| LTR | 569.316 | 146.042 | 61.769 | 0 | 0 |
| Copia | 180.685 | 275.175 | 422.246 | 471.447 | 0 |
| DIRS | 766.684 | 743.991 | 1.555.092 | 1.105.144 | 825.655 |
| ERV1 | 750.875 | 1.123.315 | 980.225 | 1.469.763 | 126.774 |
| ERVK | 659.689 | 486.486 | 0 | 513.490 | 3.133.108 |
| Gypsy | 2.510.567 | 2.056.070 | 3.675.723 | 2.213.161 | 1.672.149 |
| Ngaro | 980.001 | 3.390.987 | 850.690 | 768.211 | 292.619 |
| Pao | 419.975 | 385.357 | 723.976 | 1.151.953 | 307.558 |
| RC | 0 | 0 | 0 | 0 | 0 |
| Helitron | **6.307.248** | 3.514.054 | **4.649.952** | **5.159.806** | 2.433.280 |
| Retroposon | 213.419 | 116.255 | 421.490 | 238.385 | 0 |
| SINE | 1.210.545 | 1.637.348 | 1.401.793 | 1.524.037 | 936.353 |
| 5S-Deu-L2 | 721.151 | 965.222 | 838.622 | 0 | 873.243 |
| ID | 0 | 0 | 19.012 | 29.728 | 116.970 |
| MIR | 1.185.268 | 1.020.008 | 1.163.355 | 2.103.257 | 382.739 |
| tRNA | 3.640.638 | 3.701.722 | 3.698.003 | 3.271.022 | 3.693.238 |
| tRNA-Core | 0 | 0 | 0 | 0 | 293.857 |
| tRNA-Core-L2 | 0 | 0 | 0 | 89.805 | 106.872 |
| tRNA-Deu-L2 | 0 | 0 | 0 | 42.616 | 0 |
| tRNA-L2 | 461.844 | 0 | 103.308 | 223.271 | 301.117 |
| tRNA-V | 0 | 0 | 492.471 | 0 | 0 |
| Unknown | 149.784.304 | 121.893.498 | 134.020.433 | 144.621.637 | 113.225.894 |
| **total interspersed** | **354.992.667** | **305.241.677** | **326.860.083** | **319.430.101** | **255.255.533** |
| Low_complexity | 2.964.797 | 2.285.494 | 2.321.578 | 2.478.451 | 2.097.985 |
| Satellite | 700.110 | 1.425.777 | 1.023.206 | 656.699 | 1.718.620 |
| Simple_repeat | 30.391.090 | 32.172.324 | 29.527.186 | 34.124.020 | 27.469.062 |
| rRNA | 358.199 | 351.617 | 313.179 | 42.963 | 708.978 |
| snRNA | 0 | 0 | 0 | 9.265 | 0 |
| **Total** | **389.406.863** | **341.476.889** | **360.045.232** | **356.741.499** | **287.250.178** |
