# Supplementary material for: Multi-genome comparisons reveal gain-and-loss evolution of anti-Mullerian hormone receptor type 2 as a candidate master sex-determining gene in Percidae
Source: BMC Biol. 2024 Jun 26;22:141. doi: 10.1186/s12915-024-01935-9 (PMC11209984; doi:10.1186/s12915-024-01935-9)
Supplement: Supplementary file 10 — Additional file 10: Fig. S9. Additional gene trees for amhr2. A) Tree calculated from coding sequence. B) Tree calculated from coding plus intron sequence. C) Tree calculated from amino acid sequence. All trees share the same topology but differ in support values for some splits (SH-aLRT and UFBS tests). [file 12915_2024_1935_MOESM10_ESM.pdf]

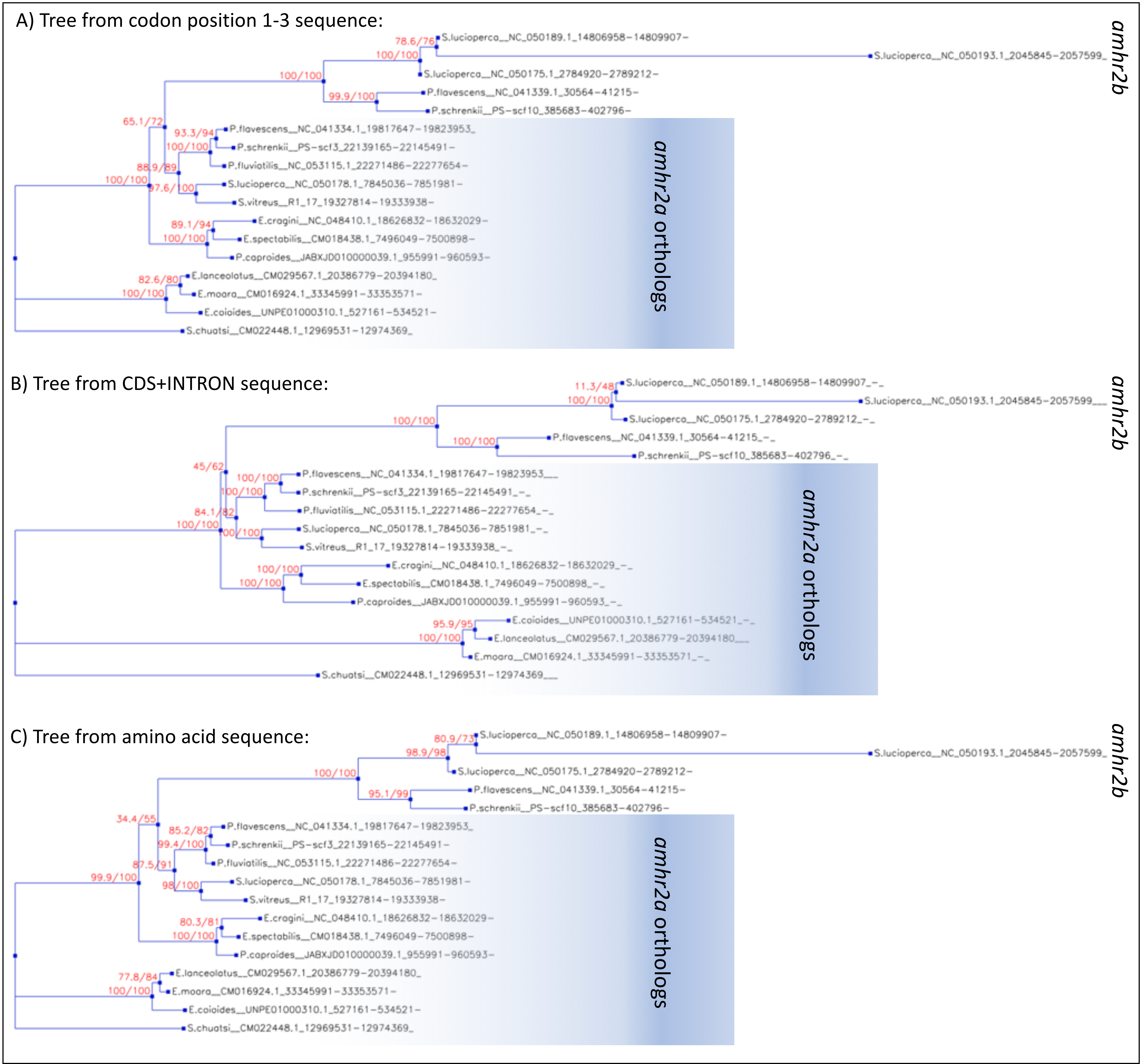

A) Tree from codon position 1-3 sequence:
amhr2b
amhr2a orthologs
S.lucioperca__NC_050189.1_14806958-14809907-
S.lucioperca__NC_050193.1_2045845-2057599_
S.lucioperca__NC_050175.1_2784920-2789212-
P.flavescens__NC_041339.1_30564-41215-
P.schrenkii__PS-scf10_385683-402796-
P.flavescens__NC_041334.1_19817647-19823953_
P.schrenkii__PS-scf3_22139165-22145491-
P.fluviatilis__NC_053115.1_22271486-22277654-
S.lucioperca__NC_050178.1_7845036-7851981-
S.vitreus__R1_17_19327814-19333938-
E.cragini__NC_048410.1_18626832-18632029-
E.spectabilis__CM018438.1_7496049-7500898-
P.caproides__JABXJD010000039.1_955991-960593-
E.lanceolatus__CM029567.1_20386779-20394180_
E.moara__CM016924.1_33345991-33353571-
E.coioides__UNPE01000310.1_527161-534521-
S.chuatsi__CM022448.1_12969531-12974369_
78.6/76
100/100
100/100
99.9/100
65.1/72
93.3/94
100/100
88.9/89
100/100
97.6/100
89.1/94
100/100
82.6/80
100/100
B) Tree from CDS+INTRON sequence:
amhr2b
amhr2a orthologs
S.lucioperca__NC_050189.1_14806958-14809907_-_
S.lucioperca__NC_050193.1_2045845-2057599__
S.lucioperca__NC_050175.1_2784920-2789212_-_
P.flavescens__NC_041339.1_30564-41215_-_
P.schrenkii__PS-scf10_385683-402796_-_
P.flavescens__NC_041334.1_19817647-19823953__
P.schrenkii__PS-scf3_22139165-22145491_-_
P.fluviatilis__NC_053115.1_22271486-22277654_-_
S.lucioperca__NC_050178.1_7845036-7851981_-_
S.vitreus__R1_17_19327814-19333938_-_
E.cragini__NC_048410.1_18626832-18632029_-_
E.spectabilis__CM018438.1_7496049-7500898_-_
P.caproides__JABXJD010000039.1_955991-960593_-_
E.coioides__UNPE01000310.1_527161-534521_-_
E.lanceolatus__CM029567.1_20386779-20394180__
E.moara__CM016924.1_33345991-33353571_-_
S.chuatsi__CM022448.1_12969531-12974369__
11.3/48
100/100
100/100
100/100
45/62
100/100
100/100
84.1/82
100/100
100/100
100/100
100/100
95.9/95
100/100
C) Tree from amino acid sequence:
amhr2b
amhr2a orthologs
S.lucioperca__NC_050189.1_14806958-14809907-
S.lucioperca__NC_050193.1_2045845-2057599_
S.lucioperca__NC_050175.1_2784920-2789212-
P.flavescens__NC_041339.1_30564-41215-
P.schrenkii__PS-scf10_385683-402796-
P.flavescens__NC_041334.1_19817647-19823953_
P.schrenkii__PS-scf3_22139165-22145491-
P.fluviatilis__NC_053115.1_22271486-22277654-
S.lucioperca__NC_050178.1_7845036-7851981-
S.vitreus__R1_17_19327814-19333938-
E.cragini__NC_048410.1_18626832-18632029-
E.spectabilis__CM018438.1_7496049-7500898-
P.caproides__JABXJD010000039.1_955991-960593-
E.lanceolatus__CM029567.1_20386779-20394180_
E.moara__CM016924.1_33345991-33353571-
E.coioides__UNPE01000310.1_527161-534521-
S.chuatsi__CM022448.1_12969531-12974369_
80.9/73
98.9/98
100/100
95.1/99
34.4/55
85.2/82
99.4/100
87.5/91
99.9/100
98/100
80.3/81
100/100
77.8/84
100/100
